# Real-time HER2 status detected on circulating tumor cells predicts different outcomes of anti-HER2 therapy in histologically HER2-positive metastatic breast cancer patients

**DOI:** 10.1186/s12885-016-2578-5

**Published:** 2016-07-25

**Authors:** Shaohua Zhang, Lei Li, Tao Wang, Li Bian, Haixu Hu, Chunhong Xu, Bing Liu, Yi Liu, Massimo Cristofanilli, Zefei Jiang

**Affiliations:** 1 Translational Medicine Center, Laboratory of Oncology, Affiliated Hospital of Academy of Military Medical Sciences, 8 Dongdajie, Beijing, 100071 China; 2 Department of Medical Oncology, Jefferson University Hospital, 1100 Walnut Street, Philadelphia, PA 19107 USA; 3 Department of Breast Cancer, Affiliated Hospital of Academy of Military Medical Sciences, 8 Dongdajie, Beijing, 100071 China

**Keywords:** Circulating Tumor Cells (CTCs), Human Epidermal Growth Factor Receptor 2 (HER2), Metastatic Breast Cancer (MBC), Anti-HER2 therapy, Real-time HER2 status

## Abstract

**Background:**

This study was initiated to investigate the difference in HER2 status between tumor tissue and circulating tumor cells (CTCs), as well as the predictive value of CTC HER2 status for predicting the outcomes of anti-HER2 therapy in histologically HER2-positive metastatic breast cancer (MBC) patients.

**Methods:**

HER2 expression on CTCs was detected using a CellSearch system within 7 days before a new line of anti-HER2 therapy was begun. According to the criterion proposed in our previous report, patients were defined as CTC HER2-positive or -negative. After close follow-up, the correlation between CTC HER2 status and the outcome of the treatment was evaluated by statistical analysis.

**Results:**

CTCs were detected in 57.4 % (58/101) of the patients. Notably, 62.1 % (36/58) of these patients had an inconsistent HER2 status between their tissue and CTCs. The discordant rate may correlate with the time interval between histological and CTC HER2 testing and is more likely to occur in the subgroup of patients with an interval of > 1 year than in those with an interval < 1 year (70.7 % vs. 41.2 %, *P* = 0.043). For PFS, positive HER2 status on CTCs was shown to be a valuable predictor, both in univariate (HR = 0.321, 95%CI, 0.156–0.62, *P* = 0.0011) and multivariate (HR = 0.383, 95%CI, 0.166–0.831, *P* = 0.019) Cox regression analysis. Meanwhile, Kaplan-Meier survival curves revealed that the median PFS of CTC HER2-positive patients was significantly longer than CTC HER2-negative ones (8.5 vs. 3.5 months, *P* < 0.001).

**Conclusions:**

HER2 status on CTCs was different from that of tumor tissues and predicted a different outcome of the patients’ anti-HER2 therapy. This difference may be correlated with the time interval between tissue and CTC HER2 testing, indicating the necessity of real-time HER2 analysis for histologically HER2-positive MBC patients.

**Electronic supplementary material:**

The online version of this article (doi:10.1186/s12885-016-2578-5) contains supplementary material, which is available to authorized users.

## Background

Human epidermal growth factor receptor 2 (HER2), also known as Erb-B2, is a well-recognized tumor marker that plays significant roles in cancer cell survival and proliferation [1]. The over-expression of this 185 kDa transmembrane tyrosine kinase receptor or the amplification of this gene located on human chromosome 17q21 results in what is called the HER2-positive molecular subtype of breast cancer that occurs in approximately 20 % of patients [2, 3] and is associated with worse outcome. The introduction of anti-HER2 therapy, such as trastuzumab, lapatinib, pertuzumab, and trastuzumab emtansine (T-DM1), has resulted in dramatic improvements in outcome of primary and metastatic disease [4–7].

Currently, the HER2 status of breast cancer patients is mainly determined by immunohistochemistry (IHC) or fluorescent in situ hybridization (FISH) on a section of tumor tissue obtained by surgery or needle biopsy. A positive result was defined by circumferential membrane staining that is complete, intense, and within > 10 % of tumor cells via the IHC method and/or HER2 copy number ≥ 6 signal/cell or a signal ratio of HER2 gene to chromosome 17 ≥ 2.0 by the FISH method [8, 9]. However, previous studies demonstrated that not all HER2-positive metastatic breast cancer (MBC) patients screened by the above 2 methods benefit from anti-HER2 therapy. The objective response rate (ORR) of single-agent trastuzumab and chemotherapy plus trastuzumab was, respectively, 26 % [10] and 50 % [11]. The ORR for chemotherapy plus lapatinib was no better at approximately 25 % [12]. Additionally, a cohort of patients with HER2-positive disease can develop disease recurrence or progression during trastuzumab or other HER-2 targeted therapy raising questions regarding the resistance mechanisms and most appropriate diagnostic modalities for treatment selection.

One possible explanation of the limited efficacy and resistance demonstrated in some patients is that the assessment results of the HER2 status may be inaccurate due to the inevitable system errors caused by tumor heterogeneity, subjectivity in result interpretation, and related factors [13]. In addition, considering that tumor cells are constantly evolving or undergoing clonal selection, and the acquisition of tumor tissue is invasive and difficult to perform dynamically, the detected HER2 status may not necessarily reflect patients’ real-time phenotypes and may therefore misinform the subsequent anti-HER2 therapy.

In contrast, circulating tumor cells (CTCs), the cancer cells that detach from the tumor and circulate in the peripheral blood as the cellular origin of metastasis, may offer a promising alternative for *real*-*time* HER2 detection with the advantages of minimal invasiveness and convenient accessibility [14–17]. Termed as a “liquid biopsy”, the enumeration and characterization of CTCs has demonstrated its clinical utility in prognosis and the prediction of therapy outcome, monitoring disease progression, and evaluating treatment responses [18–20]. With regard to HER2 in breast cancer, many research teams have made great efforts to compare the expression difference between tumor tissue and CTCs, aiming to correlate the real-time HER2 status of CTCs with patient’s response to anti-HER2 therapy [14, 21]. Nevertheless, the methods used in these studies were varied. More importantly, a widely accepted positive criterion has not yet been discovered, which has greatly hampered the application of CTC HER2 status in clinical practice.

The CellSearch^TM^ system (Veridex LLC, USA) is the only CTC assay that been approved by the Food and Drug Administration (FDA) of USA and China [18, 19]. The semi-automatic design of the machine, together with its commercialized reagent kit, eliminates the influence of man-made factors as much as possible and ensures the accuracy, reliability, and reproducibility of this system [22]. By staining the CTCs with a fluorescein isothiocyanate (FITC)-labeled anti HER2 antibody, the HER2 expression of the enriched CTCs can be successfully assessed in the fourth channel of the CellSearch™ system. Based on this technology, Pestrin et al. claimed that a patient can be defined as CTC HER2-positive when at least 50 % of the CTCs exhibit HER2 immunofluorescence (IF) signals [23]. On the contrary, some researchers recognized that each CTC’s HER2 intensity was not identical (scored as 0, 1+, 2+, or 3+) and insisted that a patient can be categorized as CTC HER2-positive if at least 1 CTC showed HER2 staining scored as 3+ [24, 25].

Though the clinical evidence for these 2 definitions was lacking, they gave us the valuable indication that a reasonable criterion for CTC HER2 positivity should take both the HER2 intensity and the percentage of CTCs with the corresponding intensity into consideration. Accordingly, a criterion set at > 30 % of CTCs over-expressing HER2 (3+) was proposed in our previous study and preliminarily verified for the first time according to the clinical outcome of anti-HER2 therapy [21]. To obtain more robust evidence, this prospective and more rationally designed study was initiated, and it’s primary and secondary endpoint was the progression-free survival (PFS) and the clinical value of our CTC HER2 positive criterion, respectively. Our results further underscore the importance and urgency of real-time HER2 testing through CTCs.

## Methods

### Study design

Histologically HER2-positive MBC patients (defined by the pathologist as IHC 3+ and/or a FISH ratio of more than 2.0) who were planned to receive a new line anti-HER2 therapy plus chemotherapy were enrolled in the present study. Eligible patients were required to have measurable or evaluable disease, with an Eastern Cooperative Oncology Group (ECOG) performance status score of 0 to 3, and with definite pathology report that described their ER/PR and HER2 status. Within 7 days before the initiation of therapy, 10 ml of peripheral blood was collected and delivered to the laboratory for CTC analysis. All treatment decisions for the patients were made according to the National Comprehensive Cancer Network (NCCN) clinical practice guidelines (Breast Cancer V.2.2011) without knowing the patients’ CTC results. The efficacy of the therapy was evaluated with computed tomography (CT) scans every 6-8 weeks until discontinuation or as clinically indicated. Responses were defined and categorized according to Response Evaluation Criteria in Solid Tumors (RECIST) 1.1. After close follow-up, the relationship between CTC HER2 expression and the outcome of anti-HER2 therapy were assessed by statistical analysis.

All patients signed an informed consent to participate in the study, which was approved by the ethics and scientific committees of the Affiliated Hospital of the Academy of Military Medical Sciences.

### CTC analysis

CTC analysis was performed with a CellSearch™ system (Veridex LLC, USA) according to the manufacturer’s instructions as described elsewhere [21]. Briefly, CTCs were immunomagnetically enriched using anti-epithelial cell adhesion molecule (EpCAM)-conjugated magnetic beads and then automatically stained with fluorescently tagged monoclonal antibodies (CD45-allophycocyanin for leukocytes and cytokeratin 8-, 18-, 19-phycoerythrin for CTCs) and nucleic acid dye 4′, 6-diamidino-2-phenylindole (DAPI). Those events with positive cytokeratin (CK), positive DAPI, negative CD45 and the appropriate cellular morphology were defined as CTCs. HER2 expression on CTCs was assessed by staining the cells with a FITC-labeled anti-HER2 antibody (Veridex LLC, USA). According to the criterion described in our previous research [21], the intensity of HER2 expression on each CTC was given a score of 0 (negative), 1+ (weak), 2+ (moderate or questionable), or 3+ (strong), and those patients with > 30 % of CTCs over-expressing HER2 (3+) were defined as CTC HER2-positive.

### Statistical analysis

For different clinical characteristics, Fisher’s exact test was used to test whether there was a significant difference between the CTC HER2-positive and-negative patients, and between CTC = 0 and CTC ≥1 patients. The rates of discordance for HER2 status in groups with different time intervals between tissue and CTC HER2 testing were compared by Chi-square test. PFS was defined as the time elapsed from the initiation of new line therapy to the documentation of disease progression (according to RECIST) or, if no progression was observed during the follow-up, to the last follow-up visit. Univariate and multivariate Cox regression analysis was used to determine the hazard ratio (HR) for the prediction of PFS. Kaplan-Meier survival curves of PFS were generated based on CTC HER2 status and compared by log-rank test. All statistical analyses were two-sided and carried out using SAS software version 9.2 (SAS Institute Inc., USA); *P* values < 0.05 were considered statistically significant.

## Results

### Patient characteristics and CTC detection

From September 2010 to November 2013, 101 histologically HER2-positive MBC patients with a median age of 48 years (range: 25 to 68) were enrolled in the present study. Those patients were divided into CTC positive and negative group, and their characteristics were shown in Additional file 1: Table S1. CTCs were detected in 57.4 % (58/101) of patients, with a mean value of 23, ranging from 1 to 323. According to our positive criterion, the 58 patients were divided into CTC HER2-positive and-negative groups. The patient characteristics of the 2 groups are listed in Table 1 and indicate that negative CTC HER2 status may easily be found in patients considered to be ER-and/or PR-positive (*P* = 0.014). In addition, no significant differences were found between the 2 groups in terms of age, metastatic number, metastatic sites, disease-free survival (DFS), or systemic therapy line and treatment option.

**Table 1:** Characteristic of Patients with CTC detection

Characteristics	Total	No. patients (%)		*P*
		CTC HER2+	CTC HER2-	
Overall	58	22 (37.9)	36 (62.1)	
Age (years)				
Median	48.5	49.0	46.5	0.289
Range	27–68	29–68	27–65	
ER and/or PR				
Positive	33	8 (24.2)	25 (75.8)	0.014
Negative	25	14 (56.0)	11 (44.0)	
No. of Metastasis				
1	8	5 (62.5)	3 (37.5)	0.238
≥ 2	50	17 (34.0)	33 (66.0)	
Metastatic sites				
Visceral	44	16 (36.4)	28 (63.6)	0.663
Non-visceral	14	6 (42.9)	8 (57.1)	
DFS				
≤ 12	24	12 (50.0)	12 (50.0)	0.111
> 12	34	10 (29.4)	24 (70.6)	
Systemic therapy line				
1	7	5 (71.4)	2 (28.6)	0.092
≥ 2	51	17 (33.3)	34 (66.7)	
Treatment option				
Trastuzumab + Chemo	43	19 (44.2)	24 (55.8)	0.096
Lapatinib + Chemo	15	3 (20.0)	12 (80.0)	

### HER2 status on CTCs

**Fig. 1:**
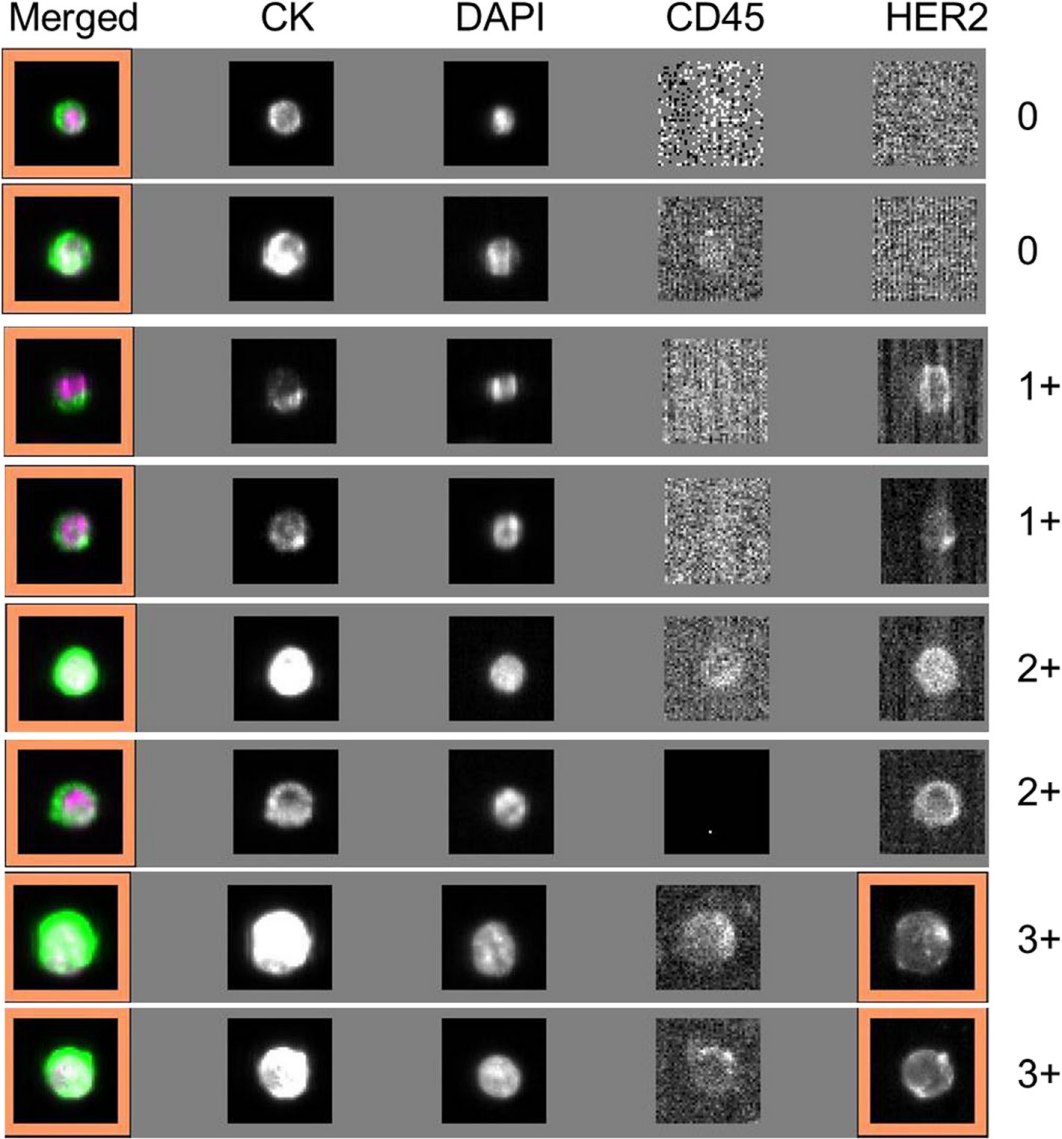
Representative images for the 0, 1+, 2+, and 3+ intensities of HER2 expression on CTCs

According to the criterion described previously, HER2 expression intensity on each CTC was given a score of 0, 1+, 2+, or 3+. Representative images are shown in Fig. 1. The percentages of CTCs at a given HER2 intensity score (0, 1+, 2+, or 3+) as well as the treatment plans for each patient are presented in Additional file 2: Table S2. Notably, with the positive criterion defined as > 30 % of CTCs with an HER2 positivity score of 3+, only 37.9 % (22/58) of patients have a consistent HER2 status between their tested tissue and CTCs. The remaining 62.1 % (36/58) of histologically HER2-positive patients were actually CTC HER2-negative. In the ER-and/or PR-positive subgroup (Luminal type), the discordant rate between tissue and CTCs reached 75.8 % (25/33). In addition, with regard to the time interval between tissue and CTC HER2 testing, the discordant rate in the subgroup of patients with an interval > 1 year was significantly higher than that of the subgroup of patients with an interval < 1 year (70.7 % vs. 41.2 %, *P* = 0.043). Similar results were also found for time intervals of 2 and 3 years, but no significant difference was found (data not shown).

### Cox regression analysis for PFS

The results of Cox regression analysis for PFS in patients with CTC detection was shown in Table 2, which revealed that positive HER2 status on CTCs was a valuable predictor for the patients’ PFS, both in univariate (HR = 0.321, 95 % CI, 0.156–0.62, *P* = 0.0011) and multivariate (HR = 0.383, 95 % CI, 0.166–0.831, *P* = 0.019) analysis. Among other characteristics, only ER and/or PR was found to significantly correlate with patient’s PFS in univariate analysis (HR = 1.854, 95 % CI, 1.015–3.454, *P* = 0.0467), but the significance disappeared in multivariate analysis (HR = 1.977, 95 % CI, 0.928–4.39, *P* = 0.0841). Considering that CTC HER2-negative patients may get less benefit from anti-HER2 therapy, the correlation between hormone receptor status and PFS may also reflect the predictive value of CTC HER2 because 75.8 % of ER- and/or PR-positive patients in our study were CTC HER2-negative (Table 1). In addition, for all enrolled patients, CTC-and CTC+ groups were considered as a variable and compared in univariate Cox regression analysis for PFS, and the results revealed that CTC number not correlated with patients’ PFS, not matter with cut-off of 1 (HR = 1.323, 95 % CI: 0.793–2.137, *P* = 0.2661) or 5 (HR = 1.329, 95 % CI: 0.849–2.102, *P* = 0.2168).

**Table 2:** Cox regression analysis for PFS predicting

Characteristics	Univariate analysis		Multivariate analysis	
	HR (95 % CI)	*P*	HR (95 % CI)	*P*
Age	0.982 (0.951–1.014)	0.2716	0.978 (0.943–1.015)	0.2371
ER and/or PR: Positive vs. Negative	1.854 (1.015–3.454)	0.0467	1.977 (0.928–4.39)	0.0841
Pathology type: ILC vs. IDC	3.911 (0.215–19.691)	0.1891	3.691 (0.169–31.259)	0.2832
Pathology type: Other vs. IDC	0.936 (0.28–2.336)	0.8995	1.49 (0.395–4.625)	0.5148
CTC Number: ≥ 5 vs. < 5	1.141 (0.631–2.064)	0.6606	1.321 (0.686–2.581)	0.4068
CTC Her2: Positive vs. Negative	0.321 (0.156–0.62)	0.0011	0.383 (0.166–0.831)	0.019
No. of Metastasis: ≥ 2 vs. = 1	0.968 (0.441–2.551)	0.9406	0.746 (0.296–2.155)	0.5566
Metastatic sites: Visceral vs. Non-visceral	0.812 (0.431–1.637)	0.5357	0.591 (0.276–1.356)	0.1907
Systemic therapy line: ≥ 2 vs. = 1	2.309 (0.84–9.543)	0.1619	2.041 (0.626–9.313)	0.2856
Treatment option: L + Che vs. T + Che	0.859 (0.446–1.789)	0.6639	0.911 (0.411–1.863)	0.8063

Abbreviation: *IDC* invasive ductal carcinoma, *ILC* invasive lobular carcinoma, *L* lapatinib, *T* trastuzumab, *Che* chemotherapy, *HR* hazard rate

### Kaplan-Meier survival curves of PFS according to CTC count and CTC HER2 Status

**Fig. 2:**
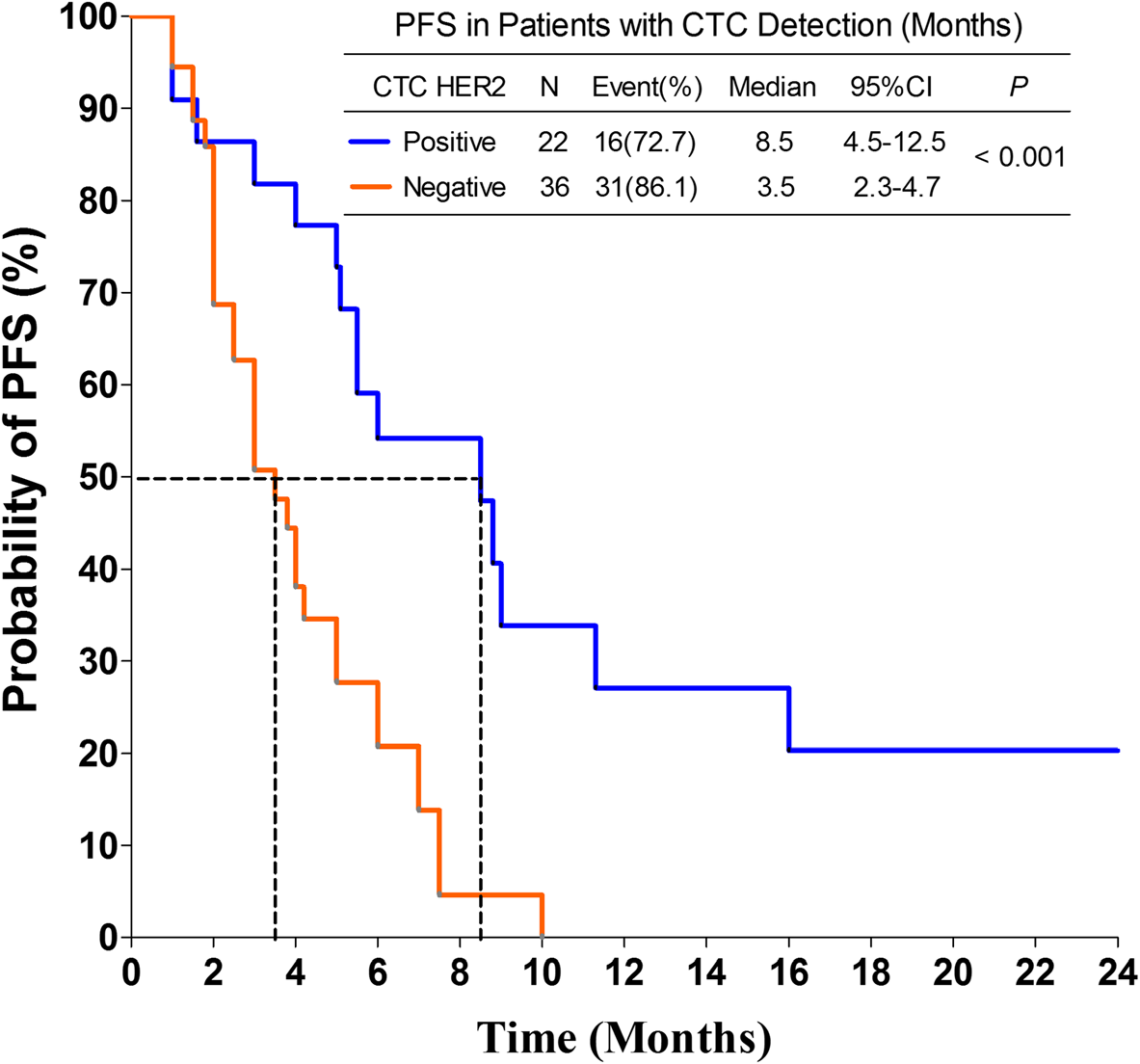
Kaplan-Meier PFS plots of CTC HER2-positive and-negative patients. PFS was calculated from the time of the baseline blood draw. The coordinates of the dashed lines indicate the median survival time

Based on CTC number, the enrolled 101 patients were divided into CTC =0 and CTC ≥ 1 groups, but the former one didn’t show superior benefit in median PFS than the latter (6.0 vs. 5.0 months, *P* = 0.2039) (Additional file 3: Figure S1). According to CTC HER2 status, 58 patients with detectable CTCs were divided into HER2-positive and -negative groups, and Kaplan-Meier survival curves to determine the PFS for the 2 groups are shown in Fig. 2. Though the 58 patients were all histologically HER2-positive and received anti-HER2 therapy, statistical analysis demonstrated that the median PFS of CTC HER2-positive patients was significantly longer than that of CTC HER2-negative patients (8.5 vs. 3.5 months, *P* < 0.001). To eliminate the potential influence of ER and/or PR status (see univariate Cox regression analysis), the 58 patients were further divided into hormone receptor-positive and-negative subgroups, among which Kaplan-Meier plots of PFS were also created according to CTC HER status. As Additional file 4: Figure S2A and S2B were shown, compared to CTC HER2-negative patients, anti-HER2 therapy significantly improved the median PFS of CTC HER2-positive patients, independent of whether they were hormone receptor-positive (5.5 vs. 3.0 months, *P* = 0.036) or -negative (8.8 vs. 5.0 months, *P* = 0.016). This finding was consistent with the results observed in all patients.

**Fig. 3:**
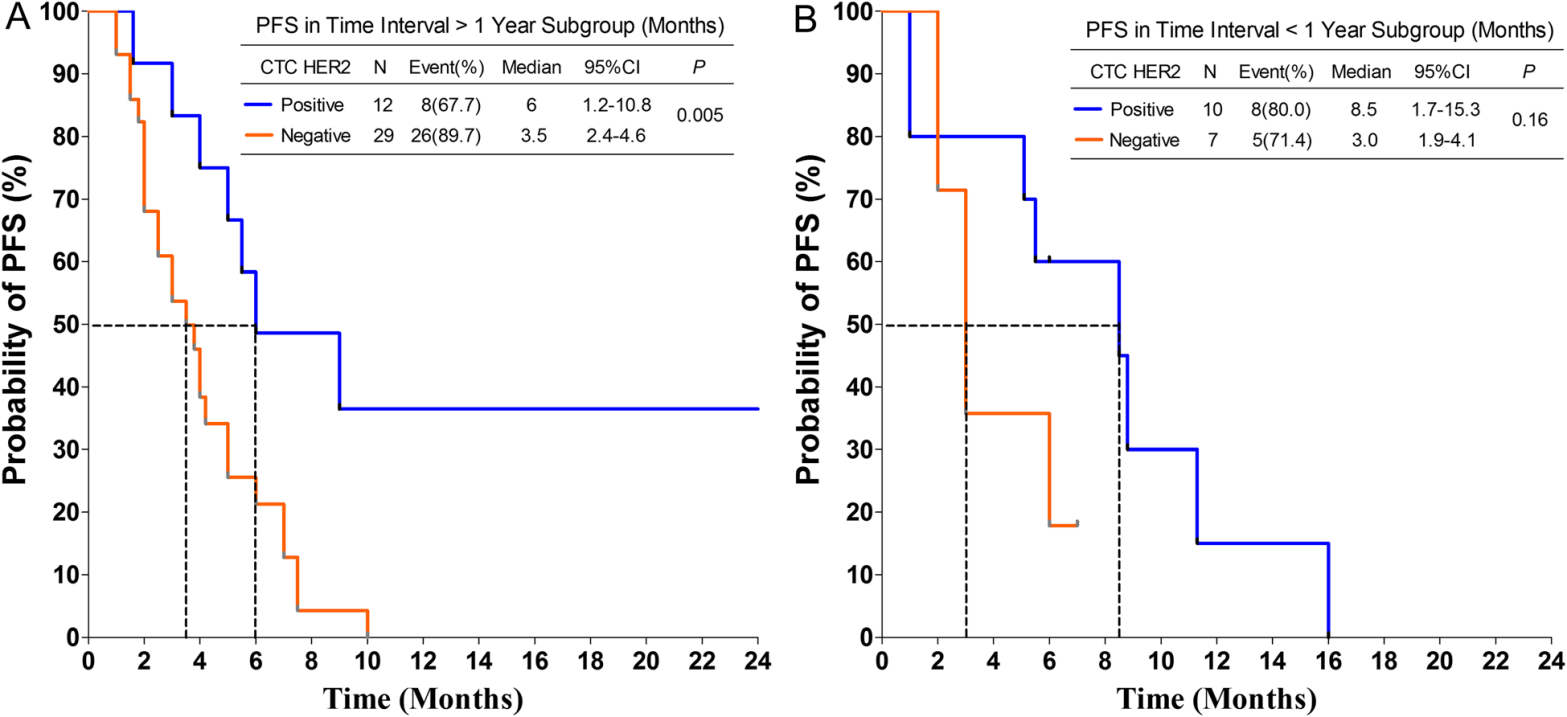
Kaplan-Meier PFS plots of CTC HER2-positive and -negative patients in subgroups of time interval (between tissue and CTC HER2 testing) > 1 year (**a**) and < 1 year (**b**). PFS was calculated from the time of the baseline blood draw. The coordinates of the dashed lines indicate median survival time

Further, the 58 patients with detectable CTC were divided into 2 subgroups according to the time interval between their tissue and CTC HER2 testing (> 1 year and < 1 year). For each subgroup, Kaplan-Meier survival curves of PFS for CTC HER2-positive and-negative patients were generated and presented in Fig. 3a and b, respectively. As the 2 figures show, although the median PFS of CTC HER2-positive patients was longer than that of HER2-negative patients, statistical significance was only found in subgroups with a time interval > 1 year (6.0 vs. 3.5 months, *P* = 0.005).

## Discussion

Growing evidence has suggested that the HER2 status of breast cancer patients may change over the course of therapy and those patients with HER2-overexpressing primary tumors may not benefit from anti-HER2 therapy if they lose HER2 expression in their metastatic sites [26, 27]. In contrast to metastatic lesions, which take time to emerge and are difficult to biopsy dynamically, the characterization of CTCs provides the real-time solution for HER2 status monitoring with the advantages of minimal invasiveness and convenient accessibility [14–16]. However, despite its promising utility, the application of CTC HER2 status testing for directing the use of anti-HER2 therapy in clinical practice has been greatly hampered due to the variety of CTC detection methods and the inconsistency in the definition of CTC HER2 positivity.

In this study, we validated definition for CTCs HER-2 positivity that derives from a series of technical considerations and takes into account the work in this field by other groups [21, 23–25]. We believe this definition reduces the subjectivity of the molecular testing necessary when proposing the clinical application of the test. In summary, we used proposed a definition for CTC HER2 positivity as > 30 % of CTCs overexpressing HER2 (3+) [21]. Unlike other proposed criteria for defining HER2 positivity in CTCs, our criterion was based on the standardized CellSearch™ system and takes into account both the intensity and percentage of CTC HER2-positive cells, emphasizing a comprehensive analysis of CTCs. In the first study, although our criterion was verified by the PFS of patients who received anti-HER2 therapy, the evidence was not strong enough due to the small sample size. In the present study, with the inclusion of a larger cohort of patients we could successfully demonstrate in univariate and multivariate Cox regression analysis the predictive value of CTC HER2 status in patients receiving anti-HER2 therapy.

Notably, the transforming of positive HER2 in tissue to negative HER2 in CTC was very common. In our present and previous study [21], the data was 62.1 % (36/58) and 51.8 % (14/27), respectively. In other literatures, though different CTC HER2 positive criterion was used, similar results were achieved, and the data of Pestrin et al., Riethdorf et al., and Ignatiadis et al. was 41.7 % (5/12) [23], 45.5 % (5/11) [24] and 50 % (1/2) [25], respectively. Such high discordant rate re-emphasized the necessity and urgency of *real*-*time* HER2 testing, especially in some developing countries (e.g., China) where anti-HER2 therapy is still not included in medical insurance and the high cost of this drug remains a great burden on patients. With the *real*-*time* characterization of CTCs, histologically HER2-positive but CTC HER2-negative patients may avoid the overtreatment with anti-HER2 therapy, which may increase health care costs (e.g., the cost of each cycle treatment of trastuzumab is approximately 24,000 RMB or 4000 $ in China) but does not necessarily have an obvious survival benefit. However, things may be different in developed countries where anti-HER2 therapy has been included in medical insurance. People don’t need to worry about the spending of anti-HER2 therapy in HER2 positive patients, but pay more attention on whether this therapy may also have function in some histologically HER2-negative but CTC HER2-positive patients. The ongoing DETECT III trial in Germany and the TREAT-CTC trail in European may provide us more evidence for this question [28].

Actually, DETECT III trial was only 1 part of the DETECT study program, which also include 3 other trials, namely, DETECT IVa, DETECT IVb and DETECT V/CHEVENDO. In each trail, different subtype of patients was enrolled and treated differentially according to the phenotypes of their CTC [29]. So far as we known, it’s the biggest prospective study that concerning about the real time analysis of CTC, more than half of the projected about 2000 patients have been enrolled. In addition to the traditional biomarkers in tissue, molecular characterization of CTC would be taken as an important indicator in this study for treatment decision making, which may make the therapy be more accurate and personalized. With the development of the study, the significance of CTC in modifying treatment option would be established. Due to this reason, the publication of its final results has attracted broad attention and was most eagerly expected.

Interestingly, CTC numbers were found not correlated with patient’s PFS in present study, not matter with cut-off of 1 or 5. This result was similar with the report of Giordano A et al. [30], but inconsistent with our previous studies [19, 21]. We presume that the reason may due to receiving or not receiving anti-HER2 therapy. In present study, only those patients who were going to receive anti-HER2 therapy were enrolled, similar with the report of Giordano A et al. However, in our previous studies, some HER2 positive patients refused the doctor’s recommendation for anti-HER2 therapy due to financial consideration. That’s the main difference between the above studies. Though it still need further verification, this observation remind us that HER2 analysis on CTC may outweigh the enumeration of CTC for histological HER2 positive patients who received anti-HER2 therapy, re-emphasizing the importance of real-time characteristic of CTC.

In addition, our results showed that the discordance between tissue and CTC HER2 status may correlate with the time interval between histological and CTC HER2 testing and is more likely to occur in the subgroups with an interval of >1 year. For tissue HER2-positive patients, the loss of HER2 may due to clonal selection under therapy [26]. Hence, it is understandable that this probability may increase along with the increased time interval between histological HER2 testing and anti-HER2 therapy. Meanwhile, with regard to median PFS, statistical significance was only found in the subgroups of patients with a time interval >1 year, indicating that the longer the time interval between tissue HER2 testing and anti-HER2 therapy, the more necessary it is to repeat tissue biopsy with assessment of the HER-2 status, but more importantly to evaluate the heterogeneity of disease by a real-time HER2 status of the patients.

One limitation of our study is that CTC data is based on one-time sampling and no additional blood draws were performed. Dynamic analysis should be warranted in the future, because it is possible that CTC count and CTC HER2 status may change over the 24-month follow-up period. In addition, it was reported that histologically HER2-positive patients may display a lower expression level of EpCAM [31] (the indispensable biomarker for CTC enrichment in CellSearch system), which may explain our finding that no CTCs were detected in 42.6 % (43/101) of the enrolled patients. Among those with detectable CTCs, 62.1 % (36/58) of them had a CTC number ranging from 1 to 10 (see Additional file 2: Table S2). We recognize that this level of CTC capturing efficiency may inevitably influence the possibility to accurately evaluate CTC HER2 status. We believe that in the future, the application of real-time HER2 testing of CTCs in clinical practice will expand also using a number of new and more sensitive and standardized CTC assays.

## Conclusions

Our study presents the prognostic value of CTC-HER2 status in patients with HER-2 positive MBC. We validated the criterion for defining CTC HER2 positivity proposed in our previous report. The discordance in HER2 status was confirmed and CTC-HER2 was proved to correlate with patients’ outcome of anti-HER2 therapy. The study reinforces the need for evaluation of molecular markers in advanced disease when possible. Future prospective studies were needed to expand on this data and focus on the identification of HER-2 positive patients that may still benefit from targeted therapies in refractory disease.

## Abbreviations

CK, cytokeratin; CT, Computed Tomography; CTC, Circulating Tumor Cells; DFS, Disease-Free Survival; EpCAM, Epithelial Cell Adhesion Molecule; FDA, Food and Drug Administration; FISH, Fluorescent In Situ Hybridization; FITC, Fluorescein Isothiocyanate; HER2, Human Epidermal Growth Factor Receptor 2; HR, Hazards Ratio; IF, immunofluorescence; IHC, immunohistochemistry; MBC, Metastatic Breast Cancer; NCCN, National Comprehensive Cancer Network; ORR, Objective Response Rate; PFS, Progression Free Survival; RECIST, Response Evaluation Criteria in Solid Tumors

## Additional files

Additional file 1: Table S1. Characteristic of the All Enrolled Patients. (DOC 56 kb) Additional file 2: Table S2. The intensity and percentage of HER2 expression on CTCs. (DOC 99 kb) Additional file 3: Figure S1. Kaplan-Meier PFS plots of CTC =0 and CTC ≥1 patients. PFS was calculated from the time of the baseline blood draw. The coordinates of the dashed lines indicate the median survival time. (JPG 548 kb) Additional file 4: Figure S2. Kaplan-Meier PFS plots of CTC HER2-positive and–negative patients in ER- and/or PR-positive subgroup (A), and the ER-and/or PR-negative subgroup (B). PFS was calculated from the time of the baseline blood draw. The coordinates of the dashed lines indicate the median survival time. (JPG 975 kb)
