# Supplementary material for: Real-time HER2 status detected on circulating tumor cells predicts different outcomes of anti-HER2 therapy in histologically HER2-positive metastatic breast cancer patients
Source: BMC Cancer. 2016 Jul 25;16:526. doi: 10.1186/s12885-016-2578-5 (PMC4960713; doi:10.1186/s12885-016-2578-5)
Supplement: Additional file 1: Table S1. — Characteristic of the All Enrolled Patients. (DOC 56 kb) [file 12885_2016_2578_MOESM1_ESM.doc]

**Supplemental Table 1** Characteristic of the All Enrolled Patients

| Characteristics | Total | No. patients (%) | | *P* |
| --- | --- | --- | --- | --- |
|  |  | CTC=0 | CTC≥1 |  |
| **Overall** | 101 | 43(42.6) | 58 (57.4) |  |
| **Age (years)** |  |  |  |  |
| Median | 48 | 48 | 48 |  |
| Range | 25-68 | 25-66 | 27-68 |  |
| **ER and/or PR** |  |  |  |  |
| Positive | 58 | 25(43.1) | 33(56.9) | **0.901** |
| Negative | 43 | 18(41.9) | 25(58.1) |  |
| **No. of Metastasis** |  |  |  |  |
| 1 | 20 | 12(60) | 8(40) | **0.078** |
| ≥ 2 | 81 | 31(38.3) | 50(61.7) |  |
| **Metastatic sites** |  |  |  |  |
| Visceral | 76 | 32(42.1) | 44(57.9) | **0.868** |
| Non-visceral | 25 | 11(44) | 14(56) |  |
| **DFS** |  |  |  |  |
| ≤ 12 | 39 | 15(38.5) | 24(61.5) | **0.507** |
| > 12 | 62 | 28(54.2) | 34(54.8) |  |
| **Systemic therapy line** |  |  |  |  |
| 1 | 19 | 12(63.2) | 7(36.8) | **0.044** |
| ≥ 2 | 82 | 31(37.8) | 51(62.2) |  |
| **Treatment option** |  |  |  |  |
| Herceptin + Chemo | 71 | 28(39.4) | 43(60.6) | **0.327** |
| Lapatinib + Chemo | 30 | 15(50) | 15(50) |  |
