# Supplementary material for: Real-time HER2 status detected on circulating tumor cells predicts different outcomes of anti-HER2 therapy in histologically HER2-positive metastatic breast cancer patients
Source: BMC Cancer. 2016 Jul 25;16:526. doi: 10.1186/s12885-016-2578-5 (PMC4960713; doi:10.1186/s12885-016-2578-5)
Supplement: Additional file 2: Table S2. — The intensity and percentage of HER2 expression on CTCs. (DOC 99 kb) [file 12885_2016_2578_MOESM2_ESM.doc]

**Supplemental Table 2** The intensity and percentage of HER2 expression on CTCs

| Patient No. | CTC count | Time interval between tissue and CTC HER2 testing (Months) | 3+  (%) | 2+  (%) | 1+  (%) | 0  (%) | PFS (Months) | Event | Therapy |
| --- | --- | --- | --- | --- | --- | --- | --- | --- | --- |
| 1 | 6 | 0 | 16.7 | 16.7 | 33.3 | 33.3 | 2 | No | Lapatinib+Capecitabine |
| 2 | 23 | 17.2 | 34.8 | 34.8 | 30.4 | 0 | 6 | Yes | Trastuzumab+Vinorelbine |
| 3 | 16 | 79.0 | 0 | 0 | 12.5 | 87.5 | 3.5 | Yes | Trastuzumab+Vinorelbine |
| 4 | 185 | 32.7 | 10.3 | 27 | 24.9 | 37.8 | 1 | Yes | Trastuzumab +Gemcitabine |
| 5 | 7 | 10.2 | 57.1 | 42.9 | 0 | 0 | 1 | Yes | Trastuzumab +Gemcitabine |
| 6 | 5 | 5.4 | 0 | 20 | 20 | 60 | 3 | Yes | Trastuzumab +Taxane+ Capecitabine |
| 7 | 1 | 84 | 0 | 100 | 0 | 0 | 4 | Yes | Trastuzumab+Vinorelbine |
| 8 | 10 | 0 | 0 | 0 | 40 | 60 | 6 | Yes | Lapatinib+Capecitabine |
| 9 | 4 | 59.4 | 50 | 25 | 0 | 25 | 6 | No | Trastuzumab +Taxane+ Capecitabine |
| 10 | 2 | 10.6 | 50 | 50 | 0 | 0 | 8.8 | Yes | Trastuzumab+Vinorelbine(+Everolimus/Placebo) |
| 11 | 3 | 72.0 | 0 | 0 | 66.7 | 33.3 | 7.5 | Yes | Trastuzumab +Taxane |
| 12 | 1 | 79.4 | 0 | 0 | 0 | 100 | 7.5 | Yes | Trastuzumab+Vinorelbine |
| 13 | 24 | 0.3 | 54.2 | 25 | 16.7 | 4.2 | 5.5 | Yes | Trastuzumab +Capecitabine |
| 14 | 1 | 93.2 | 100 | 0 | 0 | 0 | 6 | No | Trastuzumab +Gemcitabine |
| 15 | 7 | 15.1 | 28.5 | 42.9 | 14.3 | 14.3 | 3 | No | Lapatinib+Vinorelbine |
| 16 | 12 | 0.1 | 50 | 25 | 16.7 | 8.3 | 5.5 | Yes | Trastuzumab +Taxane+ Capecitabine |
| 17 | 2 | 14.5 | 0 | 0 | 50 | 50 | 1 | Yes | Trastuzumab +Gemcitabine |
| 18 | 4 | 14.2 | 0 | 25 | 50 | 25 | 7 | Yes | Lapatinib+Capecitabine |
| 19 | 38 | 29.1 | 63.2 | 15.8 | 15.8 | 23.7 | 5.5 | No | Trastuzumab +Gemcitabine |
| 20 | 28 | 1.0 | 42.8 | 14.3 | 28.6 | 14.3 | 8.5 | Yes | Trastuzumab +Taxane |
| 21 | 19 | 23.1 | 31.6 | 15.8 | 52.6 | 0 | 5 | Yes | Trastuzumab+Vinorelbine |
| 22 | 1 | 34.3 | 0 | 0 | 100 | 0 | 2 | Yes | Lapatinib+Vinorelbine |
| 23 | 2 | 6.9 | 0 | 0 | 0 | 100 | 2 | Yes | Lapatinib+Capecitabine |
| 24 | 3 | 1.8 | 33.3 | 0 | 66.7 | 0 | 5.1 | Yes | Trastuzumab +Capecitabine |
| 25 | 13 | 0 | 38.5 | 15.4 | 38.5 | 7.7 | 6 | No | Trastuzumab+Vinorelbine |
| 26 | 52 | 31.8 | 3.8 | 36.5 | 48.1 | 11.5 | 4.2 | Yes | Trastuzumab+Vinorelbine |
| 27 | 4 | 53.5 | 50 | 25 | 0 | 25 | 36 | Yes | Trastuzumab +Taxane(+Everolimus/Placebo) |
| 28 | 4 | 29.8 | 25 | 0 | 50 | 25 | 3.8 | Yes | Trastuzumab +Gemcitabine |
| 29 | 32 | 34.2 | 46.9 | 28.1 | 9.4 | 15.6 | 29 | No | Lapatinib+Capecitabine |
| 30 | 2 | 0.4 | 50 | 50 | 0 | 0 | 1 | Yes | Lapatinib+Capecitabine |
| 31 | 1 | 17.4 | 0 | 100 | 0 | 0 | 10 | Yes | Lapatinib+Capecitabine |
| 32 | 4 | 23.1 | 0 | 25 | 50 | 25 | 1.5 | Yes | Trastuzumab+Vinorelbine |
| 33 | 8 | 33.4 | 25 | 0 | 62.5 | 12.5 | 4 | Yes | Trastuzumab +Taxane |
| 34 | 1 | 30.0 | 0 | 0 | 0 | 100 | 3 | Yes | Lapatinib+Capecitabine |
| 35 | 17 | 96.8 | 35.3 | 35.3 | 23.5 | 5.9 | 3 | Yes | Trastuzumab +Gemcitabine |
| 36 | 2 | 0.1 | 50 | 50 | 0 | 0 | 16 | Yes | Lapatinib+Capecitabine |
| 37 | 2 | 13.1 | 50 | 0 | 0 | 50 | 26.5 | No | Trastuzumab +Taxane |
| 38 | 5 | 64.9 | 0 | 0 | 20 | 80 | 36 | Yes | Trastuzumab +Gemcitabine |
| 39 | 19 | 39.7 | 0 | 0 | 15.8 | 84.2 | 7 | No | Trastuzumab +Gemcitabine |
| 40 | 12 | 30.2 | 8.3 | 33.3 | 33.3 | 25 | 3 | No | Trastuzumab +Capecitabine |
| 41 | 20 | 33.8 | 5 | 10 | 55 | 30 | 5 | No | Trastuzumab+Vinorelbine |
| 42 | 29 | 5.0 | 27.6 | 31.1 | 24.1 | 17.2 | 7 | Yes | Trastuzumab +Capecitabine |
| 43 | 2 | 25.0 | 100 | 0 | 0 | 0 | 9 | No | Trastuzumab+Vinorelbine |
| 44 | 1 | 24.9 | 0 | 0 | 0 | 100 | 2.5 | Yes | Trastuzumab+Vinorelbine(+Everolimus/Placebo) |
| 45 | 2 | 61.0 | 50 | 0 | 0 | 50 | 1.6 | Yes | Trastuzumab+Vinorelbine |
| 46 | 11 | 0 | 18.2 | 54.5 | 18.2 | 9.1 | 3 | Yes | Trastuzumab +Taxane |
| 47 | 3 | 81.6 | 0 | 0 | 66.7 | 33.3 | 1.8 | Yes | Lapatinib+Capecitabine |
| 48 | 159 | 0.2 | 39 | 31.5 | 26.4 | 3.1 | 11.3 | Yes | Trastuzumab +Capecitabine |
| 49 | 86 | 67.4 | 0 | 1.2 | 16.3 | 82.6 | 5 | Yes | Trastuzumab +Gemcitabine |
| 50 | 55 | 19.8 | 0 | 4.6 | 29.1 | 67.3 | 2 | Yes | Trastuzumab +Capecitabine |
| 51 | 1 | 19.0 | 0 | 0 | 0 | 100 | 6 | Yes | Trastuzumab +Capecitabine |
| 52 | 1 | 28.7 | 0 | 100 | 0 | 0 | 1 | No | Lapatinib+Capecitabine |
| 53 | 3 | 6.8 | 0 | 0 | 0 | 100 | 2 | Yes | Trastuzumab +Gemcitabine |
| 54 | 1 | 90.2 | 0 | 100 | 0 | 0 | 4 | No | Trastuzumab+Vinorelbine |
| 55 | 1 | 29.9 | 100 | 0 | 0 | 0 | 4 | Yes | Lapatinib+Capecitabine |
| 56 | 34 | 43.0 | 14.7 | 38.2 | 41.2 | 5.9 | 2 | Yes | Trastuzumab+Vinorelbine |
| 57 | 323 | 69.6 | 0 | 0 | 15.5 | 84.5 | 2 | Yes | Lapatinib+Taxane |
| 58 | 1 | 39.9 | 0 | 0 | 0 | 100 | 1.5 | Yes | Lapatinib+Capecitabine |
