# Supplementary figures and images for: Real-time HER2 status detected on circulating tumor cells predicts different outcomes of anti-HER2 therapy in histologically HER2-positive metastatic breast cancer patients
Source: BMC Cancer. 2016 Jul 25;16:526. doi: 10.1186/s12885-016-2578-5 (PMC4960713; doi:10.1186/s12885-016-2578-5)

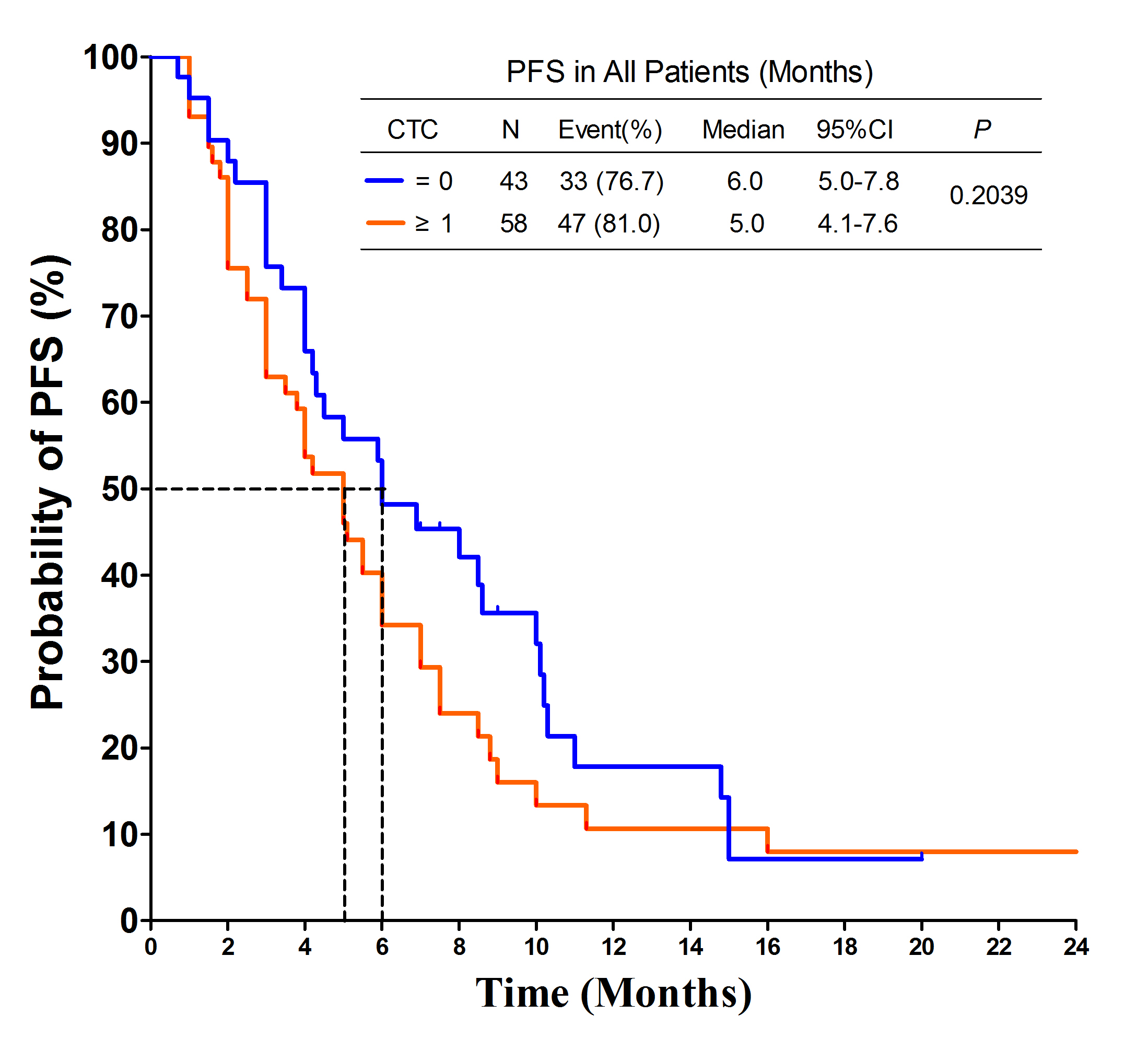

Supplement: Additional file 3: Figure S1. — Kaplan-Meier PFS plots of CTC =0 and CTC ≥1 patients. PFS was calculated from the time of the baseline blood draw. The coordinates of the dashed lines indicate the median survival time. (JPG 548 kb) [file 12885_2016_2578_MOESM3_ESM.jpg]

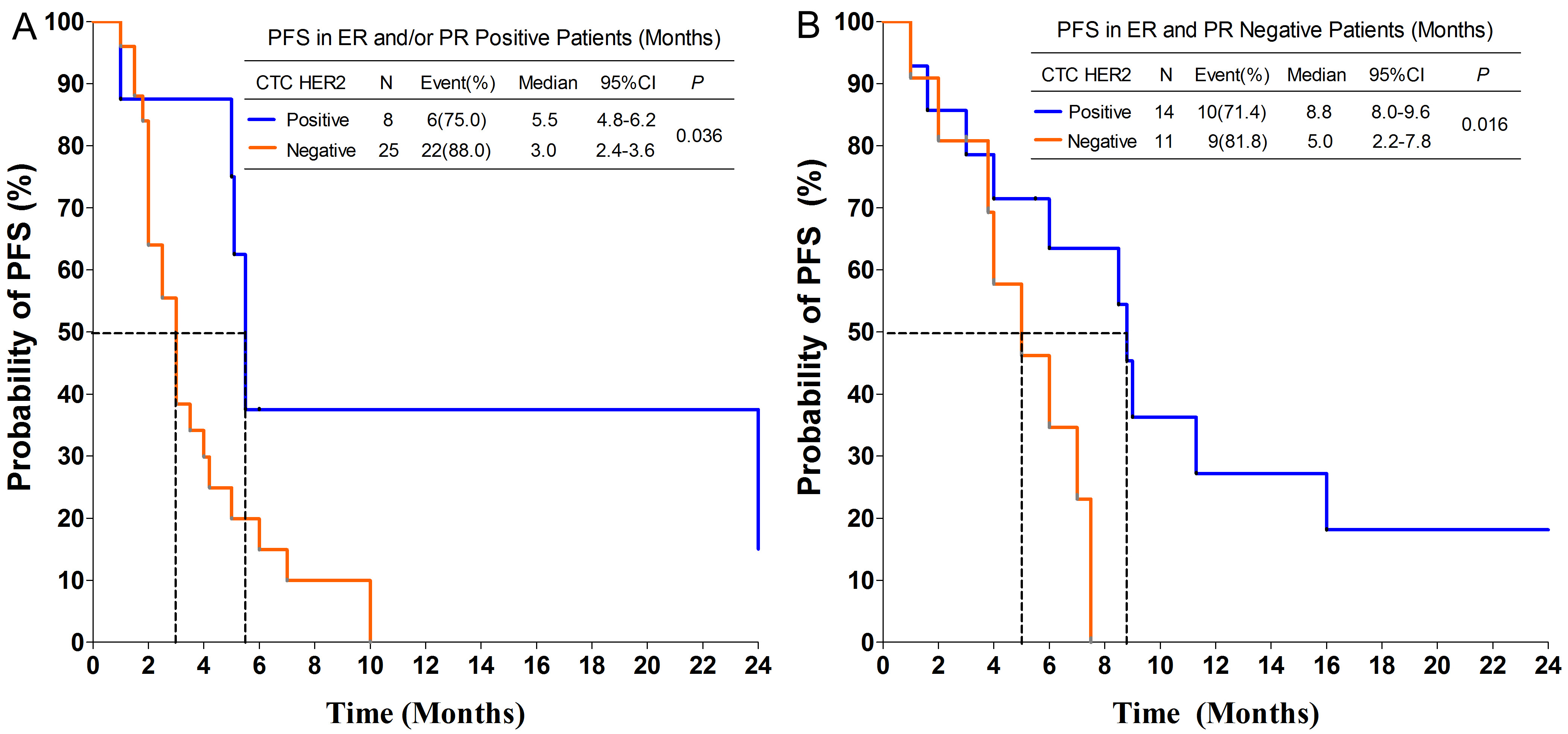

Supplement: Additional file 4: Figure S2. — Kaplan-Meier PFS plots of CTC HER2-positive and–negative patients in ER- and/or PR-positive subgroup (A), and the ER-and/or PR-negative subgroup (B). PFS was calculated from the time of the baseline blood draw. The coordinates of the dashed lines indicate the median survival time. (JPG 975 kb) [file 12885_2016_2578_MOESM4_ESM.jpg]
